# An enzyme-linked immunosorbent assay for detection of avian influenza virus subtypes H5 and H7 antibodies

**DOI:** 10.1186/1751-0147-55-84

**Published:** 2013-11-21

**Authors:** Trine H Jensen, Gitte Ajjouri, Kurt J Handberg, Marek J Slomka, Vivien J Coward, Martine Cherbonnel, Véronique Jestin, Peter Lind, Poul H Jørgensen

**Affiliations:** 1 Department of Poultry, Fish and Fur Animals, National Veterinary Institute, Technical University of Denmark, Hangøvej 2, Aarhus DK-8000, Denmark; 2 Department of Virology, Animal Health and Veterinary Laboratories Agency, AHVLA, Woodham Lane, Surrey UK-KT15 3NB, UK; 3 Anses Ploufragan Plouzané Laboratory, avian and rabbit virology immunology and parasitology unit, Ploufragan 22 440, France; 4 Department of Veterinary Epidemiology, National Veterinary Institute, Technical University of Denmark, Bülowsvej 27, Frederiksberg DK-1870, Denmark; 5 Department of Biotechnology, Chemistry and Environmental Engineering, Faculty of Engineering and Science/Aalborg Zoo, Sohngaardsholmsvej 57, Aalborg DK-9000, Denmark

**Keywords:** Avian influenza, Monoclonal antibody, Inhibition ELISA, H5, H7, Serology, Haemagglutination inhibition test, Experimental sera

## Abstract

**Background:**

Avian influenza virus (AIV) subtypes H5 and H7 attracts particular attention because of the risk of their potential pathogenicity in poultry. The haemagglutination inhibition (HI) test is widely used as subtype specific test for serological diagnostics despite the laborious nature of this method. However, enzyme-linked immunosorbent assays (ELISAs) are being explored as an alternative test method.

H5 and H7 specific monoclonal antibodies were experimentally raised and used in the development of inhibition ELISAs for detection of serological response specifically directed against AIV subtypes H5 and H7. The ELISAs were evaluated with polyclonal chicken anti-AIV antibodies against AIV subtypes: H1N2, H5N2, H5N7, H7N1, H7N7, H9N9, H10N4 and H16N3.

**Results:**

Both the H5 and H7 ELISA proved to have a high sensitivity and specificity and the ELISAs detected H5 and H7 antibodies earlier during experimental infection than the HI test did. The reproducibility of the ELISA’s performed at different times was high with Pearson correlation coefficients of 0.96-0.98.

**Conclusions:**

The ELISAs are a potential alternative to the HI test for screening of large amounts of avian sera, although only experimental sera were tested in this study.

## Background

Avian influenza is an emerging global challenge regarding the potential for pandemics with severe impact on the avian health and economy, reviewed by [1]. Of special concern is the avian influenza virus (AIV) subtype H5 and H7, which have potentials to become highly pathogenic avian influenza (HPAI) [2]. The zoonotic potential of H5 and H7 infections [3,4] and the severe impact of HPAI infections for the poultry industry [5] emphasise the need for sensitive and effective diagnostic methods and surveillances to early detect low pathogenic avian influenza infections. For this purpose, many national serological surveillance programmes rely on the use of haemagglutination inhibition (HI) test [6]. However, for screening of high numbers of samples the enzyme-linked immunosorbent assay (ELISA) techniques are superior in throughput, speed and less independent of many different antigen cultures which are needed for the HI test. Several ELISAs for detection of antibodies against the AIV nucleoprotein (NP) using inactivated NP antigen [7,8], recombinant proteins [9-13] and antigens expressed in yeast [14] have been described. These ELISAs have been tested with field sera and sera from experimentally inoculated birds of a number of different avian species including chicken [7-9,11-13,15], turkey [9,13], emu [9,13], ostrich [8,9,13] and duck [7,8,10]. Additionally, commercially available kits for AIV antibody detection have been compared to the HI test and agar gel immodiffusion (AGID) test [16-20]. These kits had higher sensitivity compared to the AGID when testing duck and wild bird sera [16,19,21]. One kit had higher sensitivity compared to HI test of a number of poultry species including duck [17], while another kit had no higher sensitivity testing domestic duck sera in comparison with the HI test [19].

ELISAs targeting H7 antibodies by use of inactivated H7 antigen [22], partially purified H7N1 antigen [23] or purified recombinant H7 protein [24] have been published. The use of recombinant protein for coating the ELISA plates may avoid steric interference by the neuraminidase protein (N) [24,25]. Inactivated whole antigen is practically applicable although it can cause problems most likely related to interference with the N protein [24]. ELISA employing a H5 monoclonal antibody (mAb) and purified H5N2 virus as coating antigen has so far been described for detection of H5 antibodies in chickens during an outbreak of A/chicken/Taiwan/1209/03(H5N2) [26] and for wild aquatic birds in Italy [27]. Two promising studies of H5 ELISA also using H5 mAb was recently described for testing of chickens, turkeys and ducks [25,28].

The continuing circulation and threat of subtypes H5 and H7 AIV (reviewed in [29]) sustain an increasing demand for diagnostic tools to detect antibodies specifically against these AIV subtypes. Consequently, we developed H5 and H7 mAbs for use in ELISA and immunocytochemistry. These H5 and H7 mAbs were applied in inhibition ELISAs and evaluated with antibodies raised experimentally in SPF chickens against a number of different AIV subtypes: H1N2, H5N2, H5N7, H7N1, H7N7, H9N9, H10N4, H16N3. The mAbs recognised AIV subtypes H5 and H7 respectively, of diverse geographic regions. Furthermore, we address the question of steric hindrance of the N component by suggesting doing a secondary ELISA test with another N type as coating antigen. The ELISA proved to be more sensitive than the HI test.

## Materials and methods

### Identity and preparation of antigen for development of the ELISA

Several influenza A strains were used for production of chicken sera for development of the ELISA test and for HI test (Table 1): A/ostrich/Denmark/72429/96 (H5N2); A/chicken/Belgium/150/99 (H5N2); A/mallard/Denmark/64650/03 (H5N7); A/African starling/England/983/79 (H7N1); A/turkey/Ireland/95 (H7N7); A/mallard/Denmark/64650G4/05 (H7N7); A/knot/England/SV497/02 (H9N9); A/turkey/England/284/79 (H10N4); A/gull/Denmark/48110/02 (H16N3) and A/swine/Denmark/13608/04 (H1N2). Avian paramyxovirus (APMV)-8/goose/Delaware/1053/76 was used to obtain AIV negative control serum. Except for the Danish avian influenza isolates [30,31] the strains were kindly supplied by the EU Reference Laboratory for Avian Influenza AHVLA, Weybridge, UK (EURL).

**Table 1:** Avian influenza strains used for raising antibodies in chickens

**Antigen**	**Origin**	**Animal**	**Number of animals**	**Age immunisation (weeks)**	**Blood samples weeks after 1. imm.**
H5N2	VET	SPF	15	3	1, 2, 3, 4, 6
A/ostrich/Denmark/72429/96					
H5N2	VET	Broiler	15	3	1, 2, 2½, 3, 6
A/ostrich/Denmark/72429/96					
H5N2	EURL	SPF	15	3, 5, 7	1, 2, 3, 4, 6
A/chicken/Belgium/150/99					
H5N7	VET	SPF	15	3, 5, 7	1, 2, 3, 4, 6
A/mallard/Denmark/64650/03					
H7N1	EURL	SPF	17	3, 5, 7	1, 2, 3, 4, 6
A/African starling/England/983/79					
H7N1	EURL	Broiler	10	3, 5, 7	1, 2, 3, 4, 6
A/African starling/England/983/79					
H7N7	EURL*	SPF	15	3, 5, 7	1, 2, 3, 4, 6
A/turkey/Ireland/95					
H9N9	EURL*	SPF	14	3, 5, 7	1, 2, 3, 4, 6
A/knot/England/02					
H10N4	EURL	SPF	16	3, 5, 7	1, 2, 3, 6
A/turkey/England/384/79					
H16N3	VET	SPF	12	4, 6, 8	1, 2, 3, 4, 5
A/gull/Denmark/7468110/02					
PMV	VET	SPF	13	3, 5, 7	1, 3, 5
APMV-8/goose/Delaware/1053/76					
H1N2	VET	SPF	15	3, 5, 7	1, 2, 3, 4, 6
A/swine/Denmark/13608/04					

*received inactivated and used directly.

imm. - immunisation.

VET- National Veterinary Institute, Technical University of Denmark.

EURL- EU Reference Laboratory for Avian Influenza, Virology Department, AHVLA Weybridge, UK.

SPF- Specific pathogen free.

Broiler- commercial broiler chickens.

The virus was propagated by inoculation in the allantoic cavity of 8-10 days old specific pathogen free (SPF) embryos (Lohmann Tierzucht, Cuxhaven, Germany). Eggs were candled daily and allantoic fluid was harvested from dead embryos. The virus was inactivated by addition of 1:1,200-2,000 β-propiolactone (Acros Organics, Geel, Belgium) to the harvest. Inactivation was confirmed by 3 blind passages in SPF eggs.

### Production and characterisation of H5 and H7 mAb

The mAb specific for the H5 (Hyb 355-02) was produced by immunisation of female Balb/c mice with sucrose purified H5N2 influenza virus A/chicken/Belgium/150/99 (H5N2). All animal experiments were conducted according to and approved by the Danish Animal Care and Ethics Committee.

The mAb specific for the H7 (Hyb 351-01) was produced by immunisation of female Balb/c mice with DNA plasmid (pCMV-HA) [32] (kindly provided by Anses, Ploufragan-Plouzané Laboratory, France). The H5 mAb was of IgG_1_ subtype and the H7 mAb was of IgG subtype while characterised using Mouse MonoAB ID kit (Zymed, California, USA) according to the manufactures protocol.

The specificity of the H5 and H7 mAbs was evaluated by direct ELISA and immunocytochemistry using 44 AIV strains belonging to 24 AIV subtypes (Table 2). The direct ELISAs to test for cross-reactivity of the H5 and H7 mAb were performed with ELISA plates coated with the different AIV antigens diluted according to their HA titer: A/duck/Alberta/35/76 (H1N1), A/chicken/Scotland/59 (H5N1), A/chicken/Belgium/150/99/ (H5N2), A/turkey/Ireland/83 PD1744/83 (H5N8), A/turkey/Ontario/7732/66 (H5N9), A/chicken/Netherlands/2992/17/03 (H7N7), A/turkey/England/384/79 (H10N4). Subsequently, the plates were incubated with mAbs diluted and subsequently processed as described for the inhibition ELISA in this article. The immunocytochemistry was done on chicken embryo fibroblast (CEF) infected with the different AIV strains (Table 2). The CEFs were fixed in 3% formaldehyde in phosphate buffered saline (PBS) and the immunocytochemistry was done as described previously [33]. 3-Amino-9-Ethylcarbazole (AEC, DAKO Cytomation, Denmark) were used to visualise the reaction between infected cells and mAbs according to standard procedures (DAKO Cytomation).

**Table 2:** Pattern of reactivity of H5 and H7 mAb against avian influenza viruses

Avian influenza isolates used for IPX and ELISA*	**Monoclonal antibodies**	
	**H5**	**H7**
H7N1 A /duck/Taiwan/98 LP	nd	+
H7N1 A/chicken/Italy/99 LP	nd	+
H7N1 A/ostrich/South_Africa/91 LP	nd	+
H7N1 A/duck/Denmark/08 LP	-	+
H7N2 A/chicken/Wales/07 LP	-	+
H7N3 A/chicken/Pakistan/95 HP	nd	+
H7N3 A/chicken/Chile/02 HP	-	+
H7N3 A/chicken/British _Columbia/514/04 HP	-	+
H7N3 A/chicken/England/06 LP	-	+
H7N3 A/chicken/Saskatchewan/07 HP	-	+
H7N7 A/chicken/Netherlands/03 HP	nd	+
H7N7 A/turkey/Ireland/98 LP	nd	+
H7N7 A/turkey/England/08 HP	-	+
H5N1 A/turkey/Turkey/05 HP	nd	-
H5N2 A/chicken/France/03 LP	nd	-
H5N2 A/mallard/Denmark/06 LP	+	-
H5N2 A/mallard/Denmark/60347/06 LP	nd	-
H5N2 A/turkey/Italy/05 LP	+	-
H5N3 A/domestic duck/Italy/04 LP	nd	-
H5N9 A/chicken/Italy/97 LP	+	-
H1N1 A/turkey/Hungary/01	nd	-
H2N3 A/mallard/England/06	nd	-
H3N2 A/duck/Singapore/02	nd	-
H4N6 A/duck/Denmark/02	nd	-
H6N1 A/teal/7394/England/06	nd	-
H6N2 A/teal/7440/England/06	nd	-
H8N4 A/turkey/Ontario/68	nd	-
H9N2 A/mallard/England/06	nd	-
H9N2 A/chicken/Iran/99	nd	-
H10N7 A/mallard/England/06	nd	-
H10N7 A/chicken/England/01	nd	-
H11N3 A/duck/Singapore/02	nd	-
H13N6 A/herring gull/Finland/05	nd	-
H14N6 A/mallard/Gurjev/91	nd	-
H16N3 A/gull/Sweden/03	nd	-
*H1N1 A*/*duck*/*Alberta*/*35*/76*	-	nd
*H5N1 A*/*chicken*/*Scotland*/59*	+	nd
*H5N2 A*/*chicken*/*Belgium*/*150*/99*	+	nd
*H5N7 A*/*mallard*/*Denmark*/*75*-*64650*/03*	+	nd
*H5N8 A*/*turkey*/*Ireland*/83*	+	nd
*H5N9 A*/*turkey*/*Ontario*/66*	+	nd
*H7N1 A*/*African starling*/*England*/*983*/79*	-	+
*H7N7 A*/*chicken*/*Netherlands*/*2992*/*17*/03*	-	+
*H10N4 A*/*turkey*/*England*/*384*/79*	-	nd

IPX immunochemistry with immunoperoxidase test.

Uninfected CEFs were used as controls.

*tested by ELISA.

nd- not done.

### Sera

Experimentally produced polyclonal sera against H1, H5, H7, H9, H10 and H16 were obtained by immunisation of SPF chickens (Lohmann Tierzucht) with influenza A strains as listed in Table 1. In addition to the SPF chickens, commercial broilers were immunised with A/ostrich/Denmark/72429/96 (H5N2) and A/African starling/England/983/79 (H7N1), respectively.

The birds (Table 1) were immunised at the age of 3, 5 and 7 weeks intramuscularly with 0.4 ml of β- propiolactone inactivated allantoic fluid (H5 and H7) and incomplete Freunds adjuvant (DIFCO Laboratories, Detroit, Michigan) in equal amounts. The birds immunised with H1, H9, H10 and H16 were given live virus orally and into the conjunctiva at the first immunisation and subsequently immunised with inactive allantoic fluid with incomplete Freunds adjuvant at the second and third immunisation.

Negative control sera from 14 SPF chickens were tested by HI test for antibodies against H5 and H7 influenza virus, Newcastle disease virus, Egg drop syndrome virus and Infectious bronchitis virus with negative results. Additionally, sera from 13 SPF chickens immunised with APMV-8/goose/Delaware/1053/76 were used as negative controls.

### HI test

The HI test of sera was performed according to the OIE Manual [6] by use of a 2-fold sera dilution and 4 haemagglutination (HA) antigen units. Chicken red blood cells (RBCs 1%, SPF chickens, Lohmann, Germany) were used. The plates were incubated at 4°C for 30 minutes and read after tilting of the plates. The HI titre was determined as the value of the highest dilution of serum causing complete inhibition of the 4 HA units of virus. Titres < 16 were considered negative in accordance with the OIE Manual [6]. All sera were tested by HI test with a homologous inactivated virus. In addition a number of sera of each subtype were tested against H5N2, H5N7, H7N1 and H7N7 inactivated virus (data not shown).

### Inhibition ELISA

ELISA plates (MaxiSorp, Nunc, Denmark) were coated with allantoic fluid harvested from SPF eggs inoculated with A/mallard/Denmark/64650/03 (H5N7) diluted 1:250 in PBS according to the HA titer of 1:256. Coated plates were kept up to 14 days at 4°C. Before use, the plates were washed 3 times (Skan Washer 300 version B, Molecular Devices) with washing buffer (PBS with 0.05% Tween 20). The test sera were diluted 1:10 in PBS containing 1% bovine serum albumin (BSA) (A9647-100G, Sigma, Denmark). A panel of positive and negative control sera was included in parallel on each plate and all sera were tested in duplicate, 100 μl of diluted serum were added to each of 2 wells and incubated for 1 hour at room temperature (rt). Subsequently, the serum dilutions were discharged by turning the plates up-side down and 100 μl of the monoclonal antibody H5 mAb Hyb 355-02 (0.025 μg/ml in PBS + 1% BSA) were added to each well. After incubation for 1 hour at rt the plates were washed as described above and 100 μl horse-radish-peroxidase conjugated polyclonal rabbit anti-mouse IgG (P0260, DakoCytomation, Glostrup, Denmark) diluted 1:1,000 in PBS + 1% BSA were added to each well. After incubation for 1 hour at rt, the plates were washed as described above and 100 μl of 1.2-phenylen-diamine-dihydrochlorid (OPD, Kem-En-Tech Diagnostics A/S, Denmark) were applied to each well. The colour development was stopped by adding 100 μl of H_2_SO_4_ 0.5 M. The optical density (OD) value of each test well was read at 492 nm with a reference of 620 nm. The percentage of inhibition (Inh%) was calculated including the mean of the OD values of the sera tested in duplicate (OD sample) and the mean of the maximum OD values for the negative control wells only containing PBS (ODmax):

$$\text{Inhibition}\;\%,\mathrm{Inh}\%=\frac{\mathrm{ODmax}-\mathrm{ODsample}}{\mathrm{ODmax}}\times 100$$

Similarly, ELISA plates were coated with inactivated A/African starling/England/983/79 (H7N1) diluted 1:300 in PBS and the procedure were as described above except for the use of the monoclonal antibody H7 mAB Hyb351-01 (0.025 μg/ml in PBS + 1% BSA).

Subsequently, a number of sera of different AIV subtypes were tested with H5N2 and H7N7 antigen to eliminate steric hindrance of the N component (Tables 3 and 4). Thus a system with 2 subsequent ELISAs was developed. First one ELISA with a specific antigen e.g. H5N7 for screening was performed followed by a second ELISA using another antigen e.g. H5N2 to exclude influence from steric hindrance of the N component. So for each serum, the final result was expressed as the lowest inhibition percentage given by ELISA.

**Table 3:** Results of testing of the heterologous sera in the H5 inhibition ELISAs

**Immunisation**	**PMV8 (n = 8)**	**H16N3 (n = 10)**	**H1N2 (n = 7)**	**H9N9 (n = 7)**	**H10N4 (n = 16)**	**H7N1 (n = 20)**	**H7N7 (n = 12)**
H5N7ag-ELISA	6.3 (13.5)	21.5 (31.0)	19.7 (26.7)	19.1 (27.9)	13.3 (18.3)	17.6 (26.6)	42.3 (55.8)
	16.2 (28.0)						
H5N2ag-ELISA	8.4 (11.9)	11.3 (15.1)	12.9 (18.7)	7.4 (11.7)	2.8 (12.2)	3.6 (18.6)	3.0 (17.8)
	6.0 (18.7)						
2 subsequent ELISA	5.6 (12.5)	11.3 (15.1)	12.9 (18.7)	7.4 (11.7)	2.7 (11.3)	3.6 (18.6)	3.0 (17.8)
	5.7 (18.3)						

Values are given as mean inhibition percentages and mean + 2 standard deviations (in brackets). Two subsequent ELISA implies that the first ELISA is done using the H5N7 antigen and then a secondary ELISA is performed with the H5N2 antigen. The minimum value for the two assays is used as the final result.

**Table 4:** Results of testing of the heterologous sera in the H7 inhibition ELISAs

**Immunisation**	**PMV8 (n = 7)**	**H16N3 (n = 6)**	**H1N2 (n = 11)**	**H9N9 (n = 7)**	**H10N4 (n = 13)**	**H5N7 (n = 14)**	**H5N2c (n = 11)**	**H5N2o (n = 20)**
H7N1ag-ELISA	-6.6 (10.1)	10.3 (17.3)	12.8 (22.1)	12.0 (19.6)	19.6 (38.2)	12.1 (25.7)	5.2 (11.0)	13.5 (26.1)
	11.3 (29.3)							
H7N7ag-ELISA	0.6 (6.1)	6.8 (22.2)	3.3 (17.3)	13.0 (25.7)	1.1 (13.2)	15.7 (30.2)	3.1 (12.0)	1.7 (14.6)
	4.4 (19.9)							
2 subsequent ELISA	-7.1 (7.6)	4.3 (14.7)	3.1 (16.2)	10.1 (18.1)	1.1 (13.2)	11.1 (24.0)	2.7 (10.7)	1.7 (14.4)
	3.0 (17.7)							

Values are given as mean inhibition percentages and mean + 2 standard deviations (in brackets). 2 subsequent ELISA implies that the first ELISA is done using the H5N7 antigen and then a secondary ELISA is performed with the H5N2 antigen.

All sera were tested twice in duplicate to test for reproducibility. The second test was always performed with a different batch of antigen coated ELISA plates and at least 1 month since the first test and in most cases also by different technicians.

### Statistical analysis

Calculations of mean values, standard deviations and coefficients of linear regression were done as standard descriptive procedures. For the 2-curve receiver operating characteristic (ROC) true positive sera were defined as those originating from the first blood sampling with a homologous HI-titer ≥ 16 (either H5- or H7-specific depending on the H5- or H7-based ELISA assessed respectively). True negative sera were selected as the latest blood sample of a heterologous infection provided a HI-titer ≥ 16 was evident with the respective homologous H-protein. For example serum from a chicken infected with H5N2 developed a HI titer ≥ 16 tested with H5N2 antigen was used as negative serum in the calculations for the H7 ELISA.

## Results

### Specificity of the H5 and H7 mAbs

The H5 and H7 mAbs were specific as they recognised only H5 and H7 subtype AIV strains shown by immunocytochemistry and direct ELISA coated with various AIV strains (Table 2).

### Specificity and sensitivity of the H5 inhibition ELISA

For studies of specificity heterologous sera from chickens immunised with PMV8, H16N3, H1N2, H9N9, H10N4 and H7N1 were chosen. For each chicken the final blood sample (5 or 6 weeks after immunisation, Table 1) was selected, provided this sera was positive in the HI-test (titer ≥16). When H5N7 inactivated virus were used as coating antigen the majority of these heterologous sera resulted in Inh% below 30. When H7N7 antisera were tested in the ELISA the results varied from 28 to 52 Inh%. With H5N2 virus as coating antigen low responses (below 20 Inh%) were obtained for the same sera, H1N2 sera yielding the highest mean response (12.9 Inh%) (Table 3). Means and standard deviations for the two assays and for 2 subsequent ELISAs, where the minimum value for the two assays are used as the final result are shown in Table 3. The Mean + 2 standard deviations for the 2 subsequent ELISA was 18.3 Inh%.

**Figure 1:**
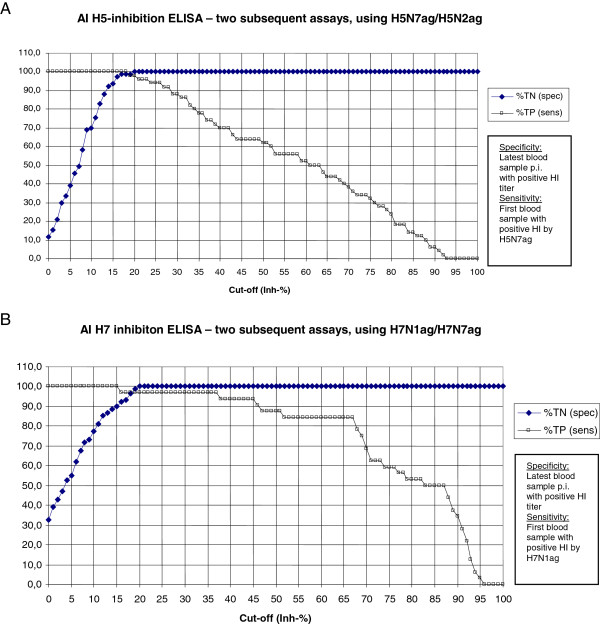
**ROC curves for inhibition ELISAs. A)** H5 ELISA and **B)** H7 ELISA. The lowest of the inhibition% (inh-%) given by each successive ELISA using H5N7ag/H5N2ag for H5, H7N1ag/H7N7ag for H7, is taken into account, Sens: sensitivity, spec: specificity, their percentage is shown in y-axis.

The first seropositive blood sample (based on HI test) from each animal were used for identification of the optimal cut-off value. ROC-curves for variable cut-off’s were produced. Using a total of 127 sera (50 true positive and 77 true negative) the 2 subsequent ELISA produced an almost perfect ROC-curve, with 98% sensitivity and 100% specificity using a cut-off at 20 Inh% (Figure 1A).

The sensitivity of the H5-ELISA was evaluated by comparison with the results of the HI-test. Sera from chickens immunised with H5N7 virus were tested using H5N2 inactivated virus as coating antigen in the ELISA and as antigen in the HI test. The H5N2 sera were also tested by both tests using inactivated H5N7 virus. Sixty sera taken at the time of immunisation (week 0) from the H5N7- and H5N2-immunised chickens were all below 20 Inh%. At weeks 1, 2 and 3 post immunisation (p.i.) sera was collected from 41 chickens. Antibodies against H5 in the serum samples were detected approximately 1 week earlier with ELISA as compared to HI test (Table 5).

**Table 5:** Comparison of detection of seroconversion of H5 and H7 antibodies with ELISA and HI test

**H5**/**H7**	**Number of animals**	**ELISA**	**HI**
Week 0	60/42	0/3.1	0/0
Week 1	41/27	54/59	2.4/11
Week 2	41/27	88/78	39/63
Week 3	41/27	98/100	93/100

The percentage of chickens which seroconverted in H5-ELISA (cut-off = 20 Inh%) and HI test (cut-off = 16), when sera were tested against heterologous N-antigen (H5N7 sera tested by ELISA plates coated with inactivated H5N2 virus and H5N2 sera tested by inactivated H5N7 antigen). After the slash are shown the percentage of chickens which seroconverted in H7-ELISA (cut-off = 20 Inh%) and HI test (cut-off = 16). Sera were tested in ELISA against heterologous N-antigen (H7N1 sera tested by ELISA plates coated with inactivated H7N7 virus and H7N7 sera tested by inactivated H7N1 virus), while all sera in HI test were tested against H7N1 inactivated virus.

### Sensitivity and specificity of the H7 inhibition ELISA

The H7 ELISA sensitivity compared to HI test was calculated the same way as for H5 (Table 5). Also in this case seroconversion was detected almost 1 week earlier with ELISA than with HI test. For the H7-ELISA, the results of ELISA with the heterologous sera are presented in Table 4. Using H7N1 inactivated virus as coating antigen in the ELISA resulted in the highest Inh% (19.6) in the sera from the H10N4 immunised chickens. In comparison coating with the inactivated H7N7 virus resulted in highest Inh% (15.7) in sera from the group of H5N7 immunised chickens. The Mean + 2 standard deviation of the 2 subsequent ELISA was 17.7 Inh%. Using a total of 121 sera (32 true positive and 89 true negative) the ROC-determination for the 2 subsequent ELISA gave a sensitivity of 97% and a specificity of 100% using a cut-off at 20 Inh% (Figure 1B).

### Stability of the inhibition ELISA

**Figure 2:**
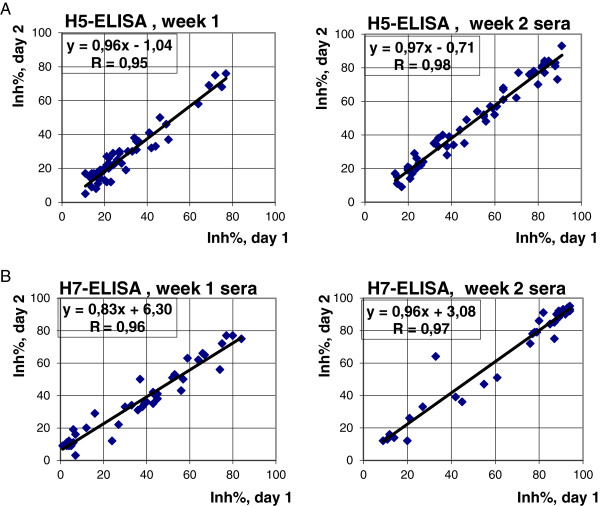
**Correlation between repeated ELISA testing on different days. (A)** H5N7 antigen and **(B)** and H7N1 antigen, Inh%: Inhibition%.

The ELISA was very stable with a good correlation between repeated ELISA tests: Pearson correlation coefficients were r = 0.96 and r = 0.98 for the H5 inhibition ELISA for sera sampled at week 1 and week 2 p.i., respectively (Figure 2A). For the H7 inhibition ELISA, the corresponding values were: r = 0.96 and r = 0.97 (Figure 2B). The coefficient of variation (standard deviation/mean) was higher for the sera sampled 1 week p.i. (14.1%) compared to sera sampled 2 weeks p.i. (7.6%). This was also the case for H7 inhibition ELISA, the coefficient of variation was 25.0% 1 week p.i. and 6.6% 2 weeks p.i.

## Discussion

The H5 and H7 mAbs developed in this study appeared to have a high specificity when tested against a variety of AIV strains in ELISA and by immune peroxidase test (Table 2). For practical reasons it was not possible to test all possible H5 and H7 subtypes but due to the high reactivity of the H5 and H7 mAbs it is assumed that these mAbs bind to conservative epitopes largely shared inside strains of the H5 and H7 subtypes respectively. Multipurpose mAbs have many advantages in diagnostic settings [12]. Since only AIV subtype H5 and H7 are reportable to the World Organization for Animal Health (OIE) and consequently the AIV surveillance normally targets two subtypes with the HI test [6], H5 and H7 mAbs were the focus of the present study.

The analysis for specificity revealed interference in the ELISA test with the N protein of the inactivated virus antigen used for coating of the ELISA plates (Tables 3 and 4). When sera raised against H7N7 virus were tested with H5N7 virus as coating antigen the result was positive (Inh% > 20) while negative (Inh% <20, Table 3) with H5N2 virus as coating antigen. H7N1 and H5N7 virus was chosen as coating antigens because these antigens gave the most optimal OD values when tested against serial dilutions of the mAb. However, the differences in OD values between the tested antigens were marginal (data not shown) and hence the ELISA is applicable for antibodies to different N-types of H5 and H7 subtype AIVs. Besides, they are LPAI viruses and were readily available. Whole inactivated virus was chosen as coating antigens because it makes the ELISA applicable in laboratories with no access to sophisticated equipment and reagents like recombinant antigens. Steric hindrance most likely could be circumvented by the use of purified or recombinant antigens for coating [23,25]. The problem with steric hindrance is known from the HI test as well [24,34].

To omit the problem for practical applicability this study suggests 2 subsequent ELISAs first using one antigen as screening followed by a secondary antigen to exclude influence from steric hindrance of the N component (Tables 3 and 4). This is in parallel to the general recommendations for AIV serological surveillance [6]. By doing 2 subsequent ELISAs the specificity are increased and the problem with interference of the N protein are omitted for both the H5 and H7 ELISA. It is suggested, based on the ROC curves (Figure 1) to define results in the first ELISA of < 20 Inh% as negative and based on the Inh% of the heterologous sera (Tables 3 and 4) an upper limit of > e.g. 80 Inh% as positive. To define such a limited window of re-testing would reduce the extra cost of performing 2 subsequent ELISAs considerably.

The H5 ELISA was shown to be able to detect antibodies one week earlier compared to the HI test (Table 5). This indicates a superior sensitivity for the performance of 2 subsequent ELISAs in the early phase of an infection with AIV H5. The same was observed with the H7 ELISA (Table 5). The higher relative sensitivity of the ELISA compared to HI test corresponds with the results of other AIV ELISAs [13,15,16,19,35-38]. Hence, it should be considered to replace the more laborious HI test or at least as initial screening in the surveillance [17,34].

Detailed analysis of the H5 and H7 mAbs used in two subsequent inhibition ELISAs resulted in a specificity of 100% for both the H5 and H7 ELISAs (Figure 1). The specificities were based on experimentally produced sera raised in chickens to homologous antigen and a variety of heterologous AIV antigens and a paramyxovirus (Tables 3 and 4). This high specificity together with the different AIV antigens used to raise the tested sera indicated that the estimates of specificity are reasonable also for field applications. However, the presence of false positives in field sera compared to experimental infections is difficult to predict, so this ELISA is a promising candidate to be evaluated using field sera from different avian species in comparison with HI test. Interestingly, it was found in another work that the HI test was most accurate in detecting antibodies of naturally compared to experimentally infected poultry [34]. Variable sensitivities and specificities have been detected by others [25] for field sera of turkeys, ducks and chicken by the use of a recombinant H5 mAb. However, others presented high sensitivities and specificities with the H5 mAb and partly purified antigens for a variety of field and experimental avian sera [8]. High sensitivities and specificities were also detected in field samples from chickens by a H5 ELISA developed during an outbreak of LPAI H5 in Taiwan [35]. Similar results were described for a H5 ELISA detecting H5 antibodies of wild ducks in Italy [27]. A H7 ELISA based on recombinant H7 mAB and inactivated antigen was shown to have higher sensitivity and specificity with experimental and field sera for use by multiple avian species compared to HI test [23]. Importantly the present H5 and H7 ELISA showed a very high degree of reproducibility (Figure 2).

## Conclusions

The inhibition ELISAs based on the H5 and H7 mAb developed in this study and a combination of two inactivated AI antigen per subtype proved to have a high sensitivity and specificity compared to HI test in experimental sera. Two AI antigens were necessary to circumvent interference with the N protein. These ELISAs detected H5 and H7 antibodies earlier during experimental infection compared to the HI test both when performed once and as 2 subsequent ELISAs. Thus the ELISAs may represent an alternative to HI test for screening for AI H5 and H7 antibodies.

## Abbreviations

AEC: 3-amino-9-ethylcarbazole; AGID: Agar gel immunodiffusion; AIV: Avian influenza virus; APMV: Avian paramyxovirus; BSA: Bovine serum albumin; CEF: Chicken embryofibroblast; EURL: EU Reference Laboratory for Avian Influenza, Virology Department, AHVLA Weybridge, United Kingdom; ELISA: Enzyme-linked immunosorbent assay; H: Haemagglutinin; HA: Haemagglutiniation; HI: Haemagglutinin inhibition; HPAI: Highly pathogenic avian influenza; Inh%: Inhibition percentage; IPX: Immunochemistry with immunoperoxidase test; mAb: Monoclonal antibody; N: Neuramindase; NP: Nucleoprotein; OP: Optical density; PBS: Phosphate buffered saline; RBC: Red blood cells; Rt: Room temperature; ROC: Receiver operating characteristic; SPF: Absence of avian adenovirus group 1, avian encephalomyelitis virus, avian infectious bronchitis virus, avian infectious laryngotracheitis virus, avian leucosis virus, avian nephritis virus, avian orthoreovirus, avian reticuloendotheliosis virus, chicken anaemia virus, egg drop syndrome virus, infectious bursal disease virus, influenza A virus, Marek’s disease virus, Newcastle disease virus, Turkey rhinotracheitis virus, mycoplasma gallisepticum, mycoplasma synoviae, salmonella pullorum; VET: National Veterinary Institute, Technical University of Denmark.

## Competing interests

The authors declare that they have no competing interest.

## Authors’ contributions

THJ was responsible for the final part of the ELISA tests and wrote the majority of the manuscript. GA developed the monoclonal antibodies and the ELISA assay. KJH participated in the design of the study and assisted in the development of the monoclonal antibodies and the setup of the ELISA experiments. VJC performed the immunocytochemistry analysis and provided antigens. MJS was involved in the design of the study and the immunocytochemistry analysis as well as providing significant numbers of the antigens. MC produced and supplied the DNA plasmid necessary for the development of the monoclonal antibody H7. VJ was involved in the study design, use of DNA plasmid and contributed to the manuscript. PL performed the statistical analysis and wrote the corresponding part of the manuscript. PHJ conceived the study, designed the study and coordinated and supervised throughout out the study. All authors have read and approved the final version of the manuscript.
